# Reactions to Pictorial and Text Cigarette Pack Warning Labels among Chinese Smokers

**DOI:** 10.3390/ijerph191811253

**Published:** 2022-09-07

**Authors:** Liu Wang, Xi Yao, Gang Wang, Kecheng Du

**Affiliations:** School of Journalism & Communication, Wuhan University, Wuhan 430072, China

**Keywords:** cigarette warning labels, graphic pictorial warnings, smoking, perceived harms, intention to quit smoking

## Abstract

**Objectives**. This study aims at examining the impact of graphic pictorial warning labels on inten-tion to quit smoking and perceived harms among Chinese working-age adults (n = 661). **Methods**. A randomized controlled trial is utilized as the research design by comparing three scenarios: Group 1 as baseline (n = 193) and presented with real market tobacco products with text-only la-bels, Group 2 as price group and with hypothetical scenarios of manipulated prices, and Group 3 as the imaging group and with hypothetical scenarios of graphic pictorial cigarette warning la-bels. Both bivariate and multivariate analyses are utilized to determine the effectiveness of warn-ing labels. **Results**. Graphic pictorial cigarette warning labels are associated with stronger inten-tion to quit smoking and greater perceived harms. Smokers have a greater number of attempts if they are less nicotine dependent and express greater smoking risk perceptions. **Conclusions**. This study adds to the evidence that graphic pictorial warning labels are more effective than text-only labels in increasing intention to quit smoking. As China strives to achieve a reduction in smoking to meet the goal of the Healthy China 2030 initiative, this work strengthens the evidence base for policy makers, practitioners, and researchers to design low-cost warning labels on cigarette packs to promote tobacco control.

## 1. Introduction

The tobacco epidemic is among the biggest public health threats in China and takes a dreadful toll on its health and economy. The recent statistics released by the World Health Organization state that there were 307.6 million smokers, accounting for 26.6% of the adult population aged 15 and above in China and one-third of the world’s total smokers [1]. The health consequence of smoking is devastating: tobacco-related diseases kill more than one million people in China each year, smoking causes twenty-three percent of all cancers in China, and the likelihood of developing chronic obstructive pulmonary disease (COPD) is 6-fold higher among male smokers than male non-smokers in China [1,2]. In addition to the health burden of tobacco-related illness and death, the economic cost of tobacco use was estimated at approximately 350 billion yuan (Chinese currency), equivalent to 57 billion US dollars in 2014 [3]. The cost is projected to grow at an unprecedented rate as there will be more than 2.2 million smoking-related deaths by 2030 in China [4].

To identify the effectiveness of cigarette package warnings among working-age adult smokers in China, this study tests three hypotheses and makes important implications. Formally, the first hypothesis tests that stronger cigarette pictorial warnings is associated with higher levels of perceived harms (H_1_). Secondly, the use of cigarette pictorial warnings is associated with stronger intention to quit smoking (H_2_). Thirdly, higher level of cigarette price is associated with stronger intention to quit smoking (H_3_). All these hypotheses are controlling for the same set of socio-demographic characteristics (age, gender, income, education, marital status, and number of children), nicotine dependence (average daily consumption of cigarettes, waiting time that passed from wakening to smoking the first cigarette of the day), and smoking risk perceptions (harming themselves and harms from exposure to second-hand smoking).

## 2. Literature Review

Increasing tobacco prices and taxes has become the dominant strategy to curb smoking and increase quit attempts across regions [5,6,7,8,9,10]. In the United States, price control of cigarette smoking is typically implemented through an excise tax on cigarettes. Using both person-level data from the 2013–2014 National Adult Tobacco Survey (NATS) and state-level data from the 1996–2013 CDC State Tobacco Activities Tracking and Evaluation (STATE), Doogan and colleagues analyzed the price-quantity model which was triggered by the federal minimum price regulation and found that a price increase led to a reduction in sales of cigarette packs [5]. In addition to federal tax, tobacco is also taxed by state and local governments. As a result of all the cigarette exercise tax rates, the higher cigarette prices decreased smoking, particularly among young males and adolescent smokers [11,12]. When facing high cigarette price, US smokers used many strategies to minimize the price effect, including choosing cheaper retail outlets and cheaper brands, using promotional offers, coupons, or discounts, identifying low-tax or non-taxed source of cigarette sellers, or purchase in large carton volumes [7,10]. This association between cigarette price increase and quantity decrease has also been observed in many other countries such as China, Japan, and Argentina [6,8,13]. Additionally, an increase in cigarette price changed smokers’ intention to quit and made them more likely to lower their level of consumption [14].

Warning labels become another strategy to encourage smoking cessation and quitting smoking. The World Health Organization Framework Convention on Tobacco Control (FCTC) treaty becomes a milestone in the promotion of public health in smoking cessation. As specified by guiding principles, people around the world have the right to live a healthy life and “Every person should be informed of the health consequences, addictive nature and mortal threat posed by tobacco consumption and exposure to tobacco smoke and effective legislative, executive, administrative or other measures should be contemplated at the appropriate governmental level to protect all persons from exposure to tobacco smoke (WHO FCTC, 2003) [15]”. Evidence of cigarette warning labels’ effectiveness is mixed and it varies across samples, study periods, and study locations. Using a sample of 3247 adults living in Beijing, Shanghai and Shenzhen in 2017, Nian and colleagues (2021) tested the perceived effectiveness and credibility of four themed pictorial health warning labels and identified that the theme of harming family or children with second-hand smoke was associated with a greater number of quit attempts [16]. In contrast, Qin and other researchers (2011) analyzed a sample of adults from Nantong and Zhangjiagang cities and found that overseas pictorial labels were effective in quit attempts but both old and new Chinese warning labels were ineffective [17]. An early study of adult smokers in Mexico found that smokers attributed a lower value to cigarette packs with graphic warning labels, which may result in a reduction in tobacco consumption [18]. Kees and colleagues (2010) assessed the influence of the 2009 Family Smoking Prevention and Tobacco Control Act and concluded that graphic images evoked fears and increased intention to quit smoking [19]. Although the warnings effect was well studied, it is worth mentioning that the FDA lost a lawsuit related to tobacco advertising and labeling restrictions and the law never went into effect [20]. The associations between pictorial warning labels and increased quit intentions were also observed in more recent empirical studies using different samples [21,22,23].

The literature is consistent in terms of the relative effectiveness of pictorial warnings and text-only warning. Early studies based on samples from US, Mexico and Canada suggested that smokers considered graphic warnings more deterrent than text-only labels [24,25]. Using the International Tobacco Control (ITC) four-country survey from Australia, Canada, the UK and USA, Borland and colleagues (2009) found that stronger imaging was able to stimulate greater cognitive and behavioral reactions and therefore increased intention to quit smoking [26]. Noar and other researchers (2015) reviewed 48 quantitative studies with independent samples and concluded that pictorial warnings were more effective than text-only warnings across eight outcomes, including intention to quit smoking as well as attitudes, beliefs, warning reactions, attention and recall [27]. Johnson and colleagues (2021) utilized randomized controlled trials of 229 adult smokers and suggested that pictorial warning labels produced stronger motivations to quit smoking and trigger emotional response [28].

This study is organized as follows. This research conducts a brief literature review of text and graphic pictorial warnings of cigarette smoking and their effectiveness in reducing smoking harms. Then, the research methodology is presented explaining the randomized controlled trial’s research design, sample participants, measurements, and analytical strategies. Additionally, empirical research findings are reported including both univariate, bivariate, and multivariate results. Lastly, conclusions are made as well as social and policy implications of all research findings. Discussions of the study limitations and directions for future research are also reported.

## 3. Materials and Methods

### 3.1. The Research Design

This study implements a randomized controlled trial (RCT) to examine the effect of graphic pictorial cigarette warning labels on intention to quit smoking and perceived harms among working-age adult smokers in China.

### 3.2. Sample Participants and Data Collection

This study includes working-age adult participants aged 19–59. Despite retirement age varying across industry and gender, people in general retire and receive pension starting at 60 years old. Total sample size is 661 participants. Group 1 has 193 participants (29.2 percent), Group 2 has 197 participants (29.8 percent), and Group 3 has 271 participants (41 percent). The inclusion criteria are: (1) working adults who earn an income; (2) aged between 19 and 59 years old; (3) current smokers. The Chinese retirement policy stipulates most men retire at sixty years old, white-collar women at 55, and blue-collar women at 50. Therefore, the working age in this study is restricted to age group 19–59 years old. This study utilizes convenience and snowball sampling methods to recruit participants. Undergraduate students from a large research university worked as research assistants on data collection and they distributed the surveys among adult residents who were recruited via their family and friends circles. The sample participants resided in twenty-eight provinces: 88 adults living in Northern China, 104 adults living in Central China, 131 in Eastern China, 29 in Southern China, and 309 in Western China. By recruiting sample participants from 28 out of 31 provinces, this study ensured the sample took into account the geographic variation in China. The survey was administered via *Wenjuanxing* (wjx.cn, accessed on 21 December 2021), an online survey platform, comparable to other widely used online tools such as *Survey Monkey* and *Qualtrics*. Respondents received the survey link and completed the questionnaire using their smart devices. The survey was collected during the COVID-19 period from November to December 2021.

This study was approved by the University’s Ethics Committee. All the student research assistants were informed of research protocols and informed consent forms were signed electronically prior to data collection. All identifiable information was discarded and the research team stored the data in a password-protected system in order to safeguard anonymity of study participants.

All those participants were randomly assigned into each of the three groups: 193 participants in Group 1 (n = 193), 197 participants in Group 2 (n = 197), and 271 participants in Group 3 (n = 271). Participants in Group 1 were presented with cigarette packs showing current market prices and trademark designs, and Group 1 was considered as the baseline control group. Participants in Group 2 were presented with cigarette packs showing current trademark designs but doubled price in a hypothetical scenario. Participants in Group 3 were presented with cigarette packs indicative of current market prices and trademark designs along with large picture-based warning labels. Groups 2 and 3 were treatment groups.

**Figure 1:**
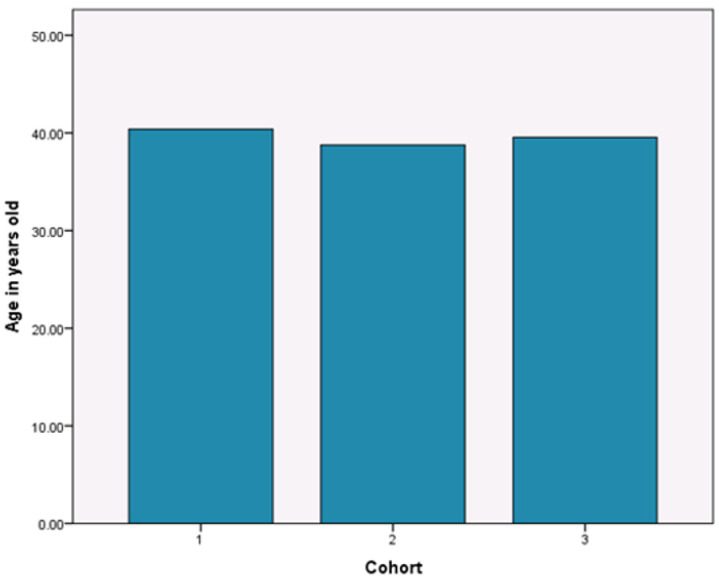
Randomness of Age Distribution.

**Figure 2:**
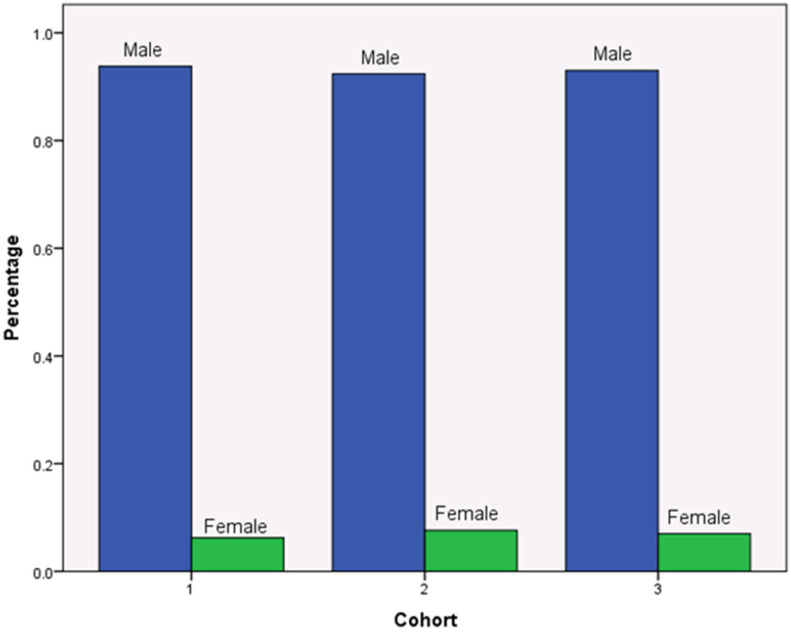
Randomness of Gender Distribution.

**Figure 3:**
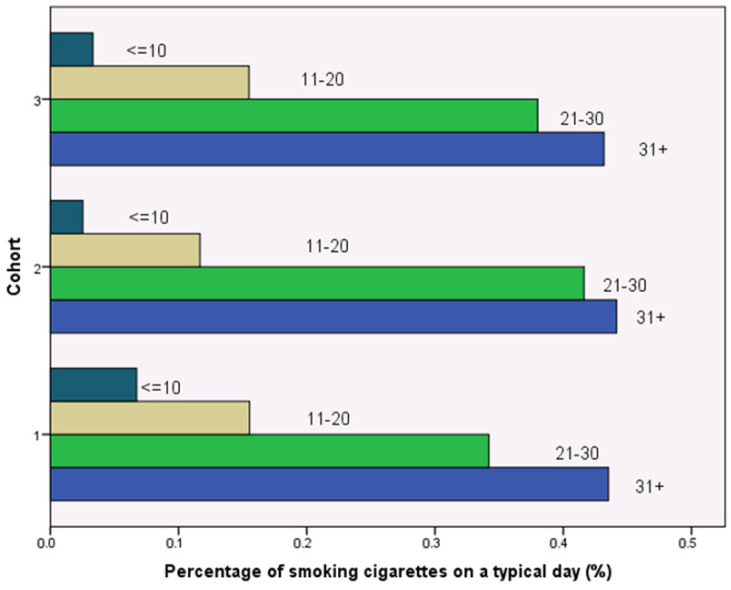
Randomness of Distribution of Average Cigarette Consumption.

**Figure 4:**
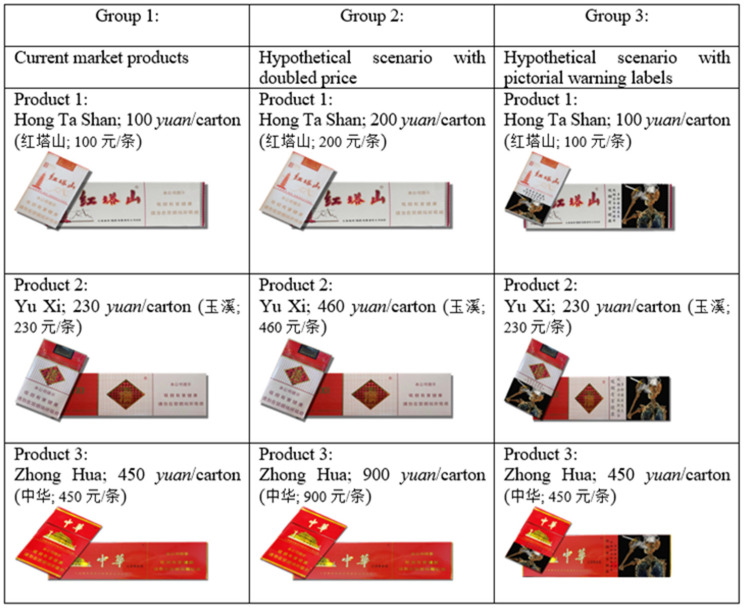
Warning Packages Applied in Survey Experiment, Including Texts and Pictures.

To ensure the randomness of group assignment, researchers utilize three factors to randomly assign subjects into control or experimental groups: age, gender, and total number of cigarettes smoked on a typical day. Statistical tests indicate the success of random assignment. As shown by Figure 1, there is no difference in terms of average ages in the three groups and participants’ mean age is 39.558 years old (SD = 10.487). The chi-square test from Table 1 reinforces that there is no statistical difference in ages among participants in the three groups (*p* = 0.319). Similar, Figure 2 presents that the gender composition is similar across the three groups, with males accounting for 93 percent and females 7 percent in each group. The chi-square test also indicates that gender distribution is consistent across the three groups (*p* = 0.863). Figure 3 indicates that the total number of cigarettes smoked in each group has an identical distribution: 43.6 percent of smokers consume ten or fewer cigarettes on a typical day, 38 percent of smokers consume 11 to 20 cigarettes daily, 14.4 percent of smokers consume 21 to 30 cigarettes daily, and 4.1 percent of smokers consume 31 and more cigarettes daily. Table 1 indicates that there is no difference in terms of percentages of smoking cigarettes on a typical day (*p* = 0.259). This random assignment assures that observable baseline characteristics of treatment groups are similar and have a balance of covariates. Figure 4 presents warning packages applied in the survey experiment. Column 1 (Group 1) lists all three products with current market prices and labels. Column 2 (Group 2) shows the hypothetical scenario with doubled market prices and labels unchanged. Column 3 (Group 3) indicates the hypothetical scenario with graphic pictorial warning labels and prices unchanged.

### 3.3. Measurement

Finally, the survey assesses socio-demographic characteristics. Age is coded in years old. Gender is dummy coded with 1 = male and 0 = female. Education level is collapsed into four categories: less than high school, completion of high school, some college, college and above. The annual income indicator is coded as four categories: 1 = below 5 wan (equivalent to USD 7500), 2 = between 60,000 and 100,000 (equivalent to USD 9000 to 15,000), 3 = between 110,000 and 200,000 (equivalent to USD 16,000 to 30,000), and 4 = more than 200,000 (equivalent to more than USD 30,000). Marital status is dichotomous by separating those who are never married from those who are either married, co-habited, divorced, or widowed. The indicator of having children is binary with 1 = yes and 0 = no. Furthermore, the extent of nicotine dependence is estimated using two 4-point indexes. The first index is measured by the average daily consumption of cigarettes: 1 = no more than ten cigarettes, 2 = between 11 and 20 cigarettes, 3 = between 21 and 30 cigarettes, 4 = 31+ cigarettes. The second index is measured by the average waiting time that passed from awakening to smoking the first cigarette of the day: 1 = no more than 5 min, 2 = between 6 and 30 min, 3 = between 31 and 60 min, 4 = more than 60 min. The higher cigarette consumption and shorter waiting time represent greater nicotine dependence. Additionally, two variables, smoking risk perception and feelings about harms associated with cigarette smoking, are measured using 4-point Likert scales. One indicator is related to harms toward themselves based on the original survey question, “In your opinion, how harmful is smoking for your health?” The other indicator is related to harms on exposure to second-hand smoke. The four response options are 1 = very little, 2 = somewhat little, 3 = somewhat major, and 4 = to a great extent.

Three dependent variables are utilized to measure intention to quit smoking. The first dependent variable, perceived harms, is based on the survey question, “To what extent do cigarette pack warnings make you think of severity of harm from smoking?” The second dependent variable, intentions of quitting smoking associated with cigarette pack warnings, is based on the survey question, “To what extent do cigarette pack warnings make you think of quitting smoking?” The third dependent variable, intentions of quitting smoking associated with cigarette price, is based on the survey question, “To what extent do cigarette prices make you think of quitting smoking?” All these variables are measured using 5-point Likert scales from 1 = strongly disagree to 5 = strongly agree.

All statistical analyses are conducted using SPSS 25.0. The chi-square test of independence reports the bivariate relationships between dependent variables and the group assignment. All the multivariate relationships are estimated using multiple linear regression models with estimates and 95 percent confidence intervals are reported. All regression models are adjusted for socio-demographic characteristics, nicotine addiction, and smoking risk perceptions. Statistical significance is determined using *p* values < 0.05, with a smaller *p*-value indicating sufficiently stronger evidence to reject the null hypothesis and accept the alternative hypothesis.

## 4. Results

### 4.1. Descriptive Statistics

Table 1 presents the descriptive statistics of the sample. The majority of the participants are male smokers (n = 615) and there are only 46 female smokers in the sample. A total of 80 participants have less than high school education (12.1 percent), 131 have high school degrees (19.8 percent), 170 have some college (25.7 percent), and 280 have at least a bachelor degree (42.4 percent). Twenty nine percent of the sample earn an annual income below 5 wan (equivalent to USD 7500), two-fifths of them earn an annual income in the range of 6–10 wan (equivalent to USD 9000–15,000), twenty percent of them earn an annual income between 11 wan and 20 wan (equivalent to USD 16,000–30,000), and nearly ten percent earn an annual income in the highest bracket(9.5 percent) with more than 20 wan (equivalent to more than USD 30,000). Three-quarters of participants are either married, co-habit, divorced or widowed, and one-quarter of them were never married. The majority of the participants have children (71.9 percent) and only 28.1 percent of them are childless.

In terms of nicotine dependence, these three subgroups do not differ from one another. On average, four-fifths of total participants consume less than one pack, twenty cigarettes a day (81.6 percent), and one-fifth of them consume more than one pack a day (18.4 percent). In terms of the average waiting time that passed from awakening to smoking the first cigarette of the day, fifteen percent of smokers to start smoking immediately after waking up, more than thirty percent of them (33.7 percent) start smoking 6 to 30 min after waking up, twenty percent of them (20 percent) start smoking 31 to 60 min after waking up, and thirty percent start smoking one hour after waking up. In comparison, these three subgroups differ from each other in terms of their perceived smoking risks. Smokers clearly understand the harmful effect of smoking on themselves: minimally harmful (6.7 percent), somewhat harmful (32.5 percent), moderately harmful (47.4 percent), and extremely harmful (13.5 percent) and the group difference is statistical significant (*p* = 0.037). Smokers perceive second-hand smoking as less harmful: minimally harmful (9.7 percent), somewhat harmful (36.8 percent), moderately harmful (40.5 percent), and extremely harmful (13.0 percent).

The mean scores of dependent variables vary substantially across three subgroups. In terms of perceived smoking harms, the distribution is: 16.2 percent of respondents strongly disagree, 11.6 percent moderately disagree, 26.8 percent are neutral, 27.2 percent moderately agree, and 18.2 percent strongly agree and the group difference is statistically significant (*p* = 0.002). In terms of intention to quit smoking associated with pack warnings, smokers follow this distribution: 16.8 percent strongly disagree, 12.6 percent moderately disagree, 34.3 percent neutral, 18.5 percent moderately agree, and 17.9 percent strongly agree and the group difference is statistically significant (*p* < 0.001). Lastly, respondents vary in terms of their intention to quit smoking associated with cigarette prices: 14.1 percent strongly disagree, 11.8 percent moderately disagree, 33.7 percent neutral, 20.1 percent moderately agree, and 20.3 percent strongly agree and three subgroups show significant differences in this regard (*p* = 0.019).

### 4.2. Multivariate Analyses

Estimates from the multivariate analyses are presented in Table 2, Table 3 and Table 4. All the multivariate analyses control the same set of socio-demographic variables, nicotine dependence, and smoking risk perceptions. Each table compares estimates from two models: Model 1 and Model 2. Model 1 is the subsample based on Group 1 and Group 2 with 390 participants, including Group 1 as the baseline group and Group 2 as the price manipulation group. Furthermore, Model 2 is the subsample based on Group 1 and Group 3 with 464 participants, including Group 1 as baseline and Group 3 as the graphic pictorial warning label group. Table 2 presents results predicting perceived harms of smoking cigarettes. Participants in Group 3 (Model 2) who reacted to graphic pictorial warning labels are more likely to perceive harms of cigarette smoking (*p* = 0.003) and this association is not observed among participants in Group 2. Additionally, Table 3 shows results predicting intention to quit. Participants in Group 3 (Model 2) have stronger intention to quit smoking after seeing the graphic depiction of the cigarette pack (*p* < 0.001) and this association is not observed among participants in Group 2. Finally, even if cigarette prices remain unchanged, participants have stronger intention to quit smoking after seeing pictorial warning labels (*p* = 0.004).

In terms of controlling variables, results from Table 2 imply that people are more likely to perceive harms of cigarette smoking if they are more educated (*p* = 0.002), less nicotine dependent (*p* = 0.008), higher risk perception of smoking harms toward themselves (*p* = 0.007). Table 3 implies that participants have stronger intention to quit if they are less nicotine dependent (*p* = 0.002) and express higher risk perception of smoking harms toward themselves (*p* = 0.008). Table 4 implies that participants with low nicotine dependent are more likely to quit smoking in future (*p* = 0.003).

## 5. Discussion

This study examines the effect of graphic pictorial warnings on cigarette packs on perceived harms and intention to quit smoking among working-age adults in China during the COVID-19 pandemic. The results indicate that pictorial warnings on cigarette packs elicited stronger quit intentions and higher level of perceived harms than text-only warnings. These findings are consistent with existing studies based on samples from other countries such as Australia, Canada, the UK, and USA [29,30,31]. Because quit intentions significantly predict actual smoking cessation behavior [32], results from this study advance our understanding of the effectiveness of pictorial cigarette warning labels during the pandemic period when smokers are more vulnerable to illnesses related to respiratory diseases including COVID-19 [33].

Nicotine dependence is negatively associated with intention to quit smoking and perceived harms. Those with higher levels of nicotine dependence are less likely to be affected by graphic pictorial warnings implying that quitting is harder for them. Because nicotine dependent smokers are likely to be chronic smokers, they become less influenced by warnings and labels. These tobacco users may demand individualized treatment plans, counseling, and medications to cope with withdrawal symptoms during their attempts to quit smoking.

Smoking risk perception is another important predictor of quit intentions. People who have stronger feelings about the harmful effects of smoking are more motivated to quit smoking. Many behavioral factors contribute to this thought process. Evoked fears may interact with smoking risk perception, leading to the intertwined effect on quit attempts. Additionally, participants who perceive harms from second-hand smoke also indicate a higher likelihood of quitting smoking.

In response to the negative health effects and economic loss of tobacco, China has made many initiatives in its tobacco control. In October 2015, China ratified the World Health Organization Framework Convention on Tobacco Control (FCTC) and the treaty came into legal force in 2016 when China started implementing regulations which prohibit sales to minors, restrict tobacco advertising, increase tobacco tax, and educate the general public on the dangers of tobacco. At the municipal level, major cities started indoor smoke bans. As of February 2021, more than twenty cities in China enacted city-level laws to prohibit smoking in indoor environments such as offices, schools, hospitals, hotels, restaurants, entertainment venues, airport terminals, and other transportation facilities [34].

The COVID-19 pandemic is an unprecedented global phenomenon and has led to enormous human loss. The most recent data in 2022 reported more than 500 million confirmed cases of COVID-19 including more than 6 million deaths worldwide [35]. The Chinese state along with the civil society has been mobilizing massive resources to combat the virus since its outbreak in January 2020. Organizations and civilians nationwide made big donations, organized food delivery and essential supplies, distributed masks, gloves, and other medical equipment in neighborhoods and communities to fight COVID-19. There were more than 5 million confirmed cases including more than twenty-three thousand deaths in China by 2022 [36]. In a world living with COVID-19, smokers become more sensitive as toxic particles spread and transmit in the air. Rodgers and colleagues (2021) reviewed forty studies in non-Chinese population settings and found that smoking, either current or past, was associated with an increased risk of COVID-19 severity [37]. The city of Chongqing, among the most important economic centers in Southwestern China, closed all the smoking rooms during the COVID-19 for the sake of residents’ health [38].

The anti-cigarette programs and services were effective as the rate of smoking in China has been declining in the past two decades. The share of male adults aged 15 years and older who smoked in China in 2000 was 30.1 percent and the prevalence rate of smoking became 24.7 percent in 2018, a significant reduction [39]. However, even though the rate of smoking has been steadily declining, China still remains the largest tobacco producer and consumer in the world. The China National Tobacco Corporation (CNTC), the world largest tobacco producer, contributes between 9 percent and 12 percent of the Chinese government’s annual state revenue [40]. Despite smoking bans in public space in many cities, smoking remains ubiquitous in China, on the street as well as inside buildings and residential homes. Unlike smokers in Western societies, Chinese smokers consider smoking as an ingrained part of socializing and tobacco use is tightly tied to Chinese culture. People purchase cigarettes as gifts for building relationships, celebrating festivals and ceremonies or many other important events. High-priced cigarette brands are considered as a symbol of high status. As China strives to achieve the Healthy China 2030 initiative, China has to face many challenges in the health system as well as health education related to healthy lifestyles and physical fitness. It is desired that China will achieve a smoke-free environment in future.

This empirical study has several methodological limitations. First, the cross-sectional design only allows this research to build relationships between socio-economic factors and smoking behaviors at one given point in time. Secondly, the sampling method is non-probability and sample size is relatively small. The use of non-probability sampling sets limitations for us to generalize this finding to a broader segment of the population, given the vast geographic and economic variations across regions in China. Third, all the outcome indicators are self-reported and may be subject to a social desirability bias. Adult respondents may respond to survey questions systematically in ways that are more socially acceptable. This bias may threaten the validity of research findings because it can overestimate the parameters of interest. Future research should aim to incorporate objective outcome indicators such as duration of smoking abstinence.

## 6. Conclusions

The results of this study advance our understanding of the effectiveness of pictorial cigarette warning labels. The present study is consistent with the literature that graphic images have stronger effects on intention to quit smoking compared to text-only warning labels. A reduction in the rate of smoking is part of the Healthy China 2030 initiative. As graphic warning labels have been proved to be an effective low-cost means of educating and informing smokers of potential harms, there is a need for future research on the evidence-based designs of such warning labels. Cigarette smoking in China is not only consumption behavior but also serves as a social currency because social norms of cigarette sharing and gifting are still ubiquitous [41]. Giving the social barriers to tobacco cessation, policy makers, practitioners, and researchers need to design more appropriate warning labels by utilizing product differentiation and market segmentation strategies such as differentiating young and new smokers versus long-term smokers. Additionally, future research must continue to explore the psychological mechanisms through which graphic imaging impacts behavior change and extend current theories such as the Health Belief Model from the 1970s [42], the Elaboration Likelihood Model from the 1980s [43], and the Theory of Planned Behavior from the 1990s [44] to make elaborate the current realities. Our results strengthen the literature by adding empirical evidence based on a sample of working-age adult smokers during the COVID-19 pandemic.

## Figures and Tables

**Table 1:** Descriptive statistics.

	Group 1	Group 2	Group 3	All Observations	Chi-Square Test *p*-Value
Dependent Variables					
Perceived harm	3.06 (1.276)	2.98 (1.321)	3.45 (1.298)	3.20 (1.314)	0.002
Intention to quit smoking due to pictorial warnings	2.83 (1.239)	2.87 (1.257)	3.41 (1.310)	3.08 (1.301)	<0.001
Intention to quit smoking due to cigarette price	3.00 (1.254)	3.15 (1.326)	3.40 (1.255)	3.21 (1.285)	0.019
Age	40.373 (10.298)	38.766 (10.636)	39.553 (10.505)	39.558 (10.487)	0.319
Gender					0.863
Male	93.8%	92.4%	93%	93.0%	
Female	6.2%	7.6%	7.0%	7.0%	
Education					<0.001
Less than HS	9.3%	6.1%	18.5%	12.1%	
High school	23.8%	14.7%	20.7%	19.8%	
Some college	31.6%	25.9%	21.4%	25.7%	
Bachelor or above	35.2%	53.3%	39.5%	42.4%	
Income					0.034
Below 5 wan	28%	22.8%	33.9%	28.9%	
Between 6 and 10 wan	39.9%	41.1%	42.1%	41.1%	
Between 11 and 20 wan	24.4%	23.9%	15.1%	20.4%	
More than 20 wan	7.8%	12.2%	8.9%	9.5%	
Marital status					0.082
Never married	18.7%	27.9%	21.8%	22.7%	
Other	81.3%	72.1%	78.2%	77.3%	
Having children					0.400
Yes	74.6%	68.5%	72.3%	71.9%	
No	25.4%	31.5%	27.7%	28.1%	
Nicotine dependence					
Average cigarette consumption					0.259
No more than 10 cigarettes	43.5%	44.2%	43.2%	43.6%	
Between 11 and 20 cigarettes	34.2%	41.6%	38.0%	38.0%	
Between 21 and 30 cigarettes	15.5%	11.7%	15.5%	14.4%	
31+ cigarettes	6.7%	2.5%	3.3%	4.1%	
Average waiting time from wakening to smoking the first cigarette					0.068
No more than 5 min	20.7%	9.6%	14.4%	14.8%	
Between 6 and 30 min	33.2%	33.5%	34.3%	33.7%	
Between 31 and 60 min	17.1%	20.3%	21.8%	20.0%	
More than 60 min	29%	36.5%	29.5%	31.5%	
Harming themselves					0.037
Very little	10.4%	4.6%	5.5%	6.7%	
Somewhat little	30.6%	38.1%	29.9%	32.5%	
Somewhat major	42.5%	44.7%	52.8%	47.4%	
To a great extent	16.6%	12.7%	11.8%	13.5%	
Harms exposure to second-hand smoke					0.746
Very little	10.4%	8.6%	10.0%	9.7%	
Somewhat little	36.3%	40.1%	34.7%	36.8%	
Somewhat major	37.8%	40.1%	42.8%	40.5%	
To a great extent	15.5%	11.2%	12.5%	13.0%	
Obs.	193	197	271	661	

Note: mean (std. dev.).

**Table 2:** Predicting Perceived Harms of Smoking Cigarettes.

	Model 1 (Group 1 vs. 2)		Model 2 (Group 1 vs. 3)	
	Coefficient (Lower to Upper 95% CI)	*p*-Value	Coefficient (Lower to Upper 95% CI)	*p*-Value
Group indicator	−0.090 (−0.355 to 0.176)	0.507	0.180 (0.064 to 0.297) **	0.003
Age	0.014 (−0.005 to 0.033)	0.137	0.013 (−0.003 to 0.028)	0.104
Gender (0/1)	0.182 (−0.351 to 0.716)	0.503	0.656 (0.182 to 1.131) **	0.007
Education				
Less than HS	0.017 (−0.519 to 0.554)	0.949	−0.598 (−0.981 to −0.214) **	0.002
High school	0.019 (−0.373 to 0.411)	0.923	0.012 (−0.315 to 0.338)	0.944
Some college	0.007 (−0.318 to 0.331)	0.969	−0.183 (−0.481 to 0.116)	0.230
BA+ (reference)				
Income				
Below 5 wan	0.304 (−0.214 to 0.821)	0.249	−0.178 (−0.649 to 0.293)	0.458
Between 6 and 10 wan	0.285 (−0.192 to 0.762)	0.241	−0.174 (−0.619 to 0.272)	0.445
Between 11 and 20 wan	0.294 (−0.202 to 0.791)	0.244	−0.535 (−1.109 to −0.050) *	0.031
>20 wan (reference)				
Marital status				
Never married	0.277 (−0.309 to 0.863)	0.353	0.489 (−0.003 to 0.965) ^†^	0.052
Other (reference)				
Having children (0/1)	0.218 (−0.333 to 0.768)	0.437	0.276 (−0.186 to 0.737)	0.241
Average daily consumption of cigarettes	−0.103 (−0.284 to 0.078)	0.263	−0.116 (−0.274 to 0.041)	0.147
Average waiting time of smoking first cigarette after waking up	0.087 (−0.051 to 0.226)	0.216	0.166 (0.043 to 0.290) **	0.008
Harming themselves	0.214 (0.001 to 0.427) *	0.049	0.249 (0.069 to 0.428) **	0.007
Harms on exposure to second-hand smoke	−0.046 (−0.254 to 0.161)	0.660	0.087 (−0.085 to 0.259)	0.321
Adjusted R-square	0.048		0.118	
N	N = 390		N = 464	

Note: ** *p* ≤ 0.01, * *p* ≤ 0.05, and ^†^ *p* ≤ 0.1.

**Table 3:** Predicting Intention to Quit Smoking with Pictorial Warning Depictions.

	Model 1 (Group 1 vs. 2)		Model 2 (Group 1 vs. 3)	
	Coefficient (Lower to Upper 95% CI)	*p*-Value	Coefficient (Lower to Upper 95% CI)	*p*-Value
Group indicator	0.064 (−0.185 to 0.313)	0.615	0.262 (0.146 to 0.378) ***	<0.001
Age	0.012 (−0.005 to 0.030)	0.162	0.012 (−0.003 to 0.028)	0.116
Gender (0/1)	0.399 (−0.101 to 0.900)	0.118	0.362 (−0.110 to 0.834)	0.133
Education				
Less than HS	0.089 (−0.415 to 0.593)	0.729	−0.306 (−0.688 to 0.076)	0.116
High school	0.001 (−0.367 to 0.369)	0.997	0.050 (−0.275 to 0.375)	0.761
Some college	−0.082 (−0.386 to 0.223)	0.599	−0.161 (−0.458 to 0.136)	0.288
BA+ (reference)				
Income				
Below 5 wan	0.329 (−0.156 to 0.815)	0.183	0.064 (−0.405 to 0.532)	0.789
Between 6 and 10 wan	0.032 (−0.416 to 0.480)	0.888	0.004 (−0.440 to 0.448)	0.986
Between 11 and 20 wan	0.080 (−0.385 to 0.546)	0.734	−0.295 (−0.777 to 0.187)	0.230
>20 wan (reference)				
Marital status				
Never married	−0.091 (−0.641 to 0.459)	0.745	0.254 (−0.227 to 0.736)	0.300
Other (reference)				
Having children (0/1)	0.108 (−0.409 to 0.624)	0.682	0.246 (−0.213 to 0.705)	0.292
Average daily consumption of cigarettes	−0.252 (−0.422 to −0.082) **	0.004	−0.072 (−0.228 to 0.085)	0.368
Average waiting time of smoking first cigarette after waking up	0.009 (−0.121 to 0.139)	0.892	0.198 (0.076 to 0.321) **	0.002
Harming themselves	0.166 (−0.033 to 0.366)	0.102	0.241 (0.062 to 0.419) **	0.008
Harms on exposure to second-hand smoke	0.095 (−0.100 to 0.290)	0.337	0.159 (−0.012 to 0.330) ^†^	0.068
Adjusted R-square	0.092		0.119	
N	N = 390		N = 464	

Note: *** *p* ≤ 0.001, ** *p* ≤ 0.01 and ^†^ *p* ≤ 0.1.

**Table 4:** Predicting Intention to Quit Smoking with Current Cigarette Prices.

	Model 1 (Group 1 vs. 2)		Model 2 (Group 1 vs. 3)	
	Coefficient (Lower to Upper 95% CI)	*p*-Value	Coefficient (Lower to Upper 95% CI)	*p*-Value
Group indicator	0.178 (−0.085 to 0.440)	0.185	0.166 (0.053 to 0.280) **	0.004
Age	0.001 (−0.018 to 0.019)	0.941	0.012 (−0.003 to 0.027)	0.104
Gender (0/1)	0.107 (−0.421 to 0.635)	0.691	0.312 (−0.149 to 0.773)	0.184
Education				
Less than HS	0.198 (−0.333 to 0.730)	0.464	−0.225 (−0.598 to 0.148)	0.236
High school	0.112 (−0.276 to 0.499)	0.572	−0.044 (−0.362 to 0.273)	0.784
Some college	−0.143(−0.465 to 0.178)	0.381	−0.344 (−0.634 to −0.053) *	0.020
BA+ (reference)				
Income				
Below 5 wan	0.448 (−0.064 to 0.960) ^†^	0.086	0.006 (−0.453 to 0.464)	0.981
Between 6 and 10 wan	0.372 (−0.101 to 0.844)	0.123	−0.068 (−0.502 to 0.365)	0.757
Between 11 and 20 wan	0.156 (−0.335 to 0.648)	0.532	−0.430 (−0.902 to 0.041) ^†^	0.073
>20 wan (reference)				
Marital status				
Never married	−0.115 (−0.695 to 0.465)	0.698	−0.020 (−0.491 to 0.451)	0.934
Other (reference)				
Having children (0/1)	0.064(−0.481 to 0.609)	0.818	0.018 (−0.430 to 0.467)	0.936
Average daily consumption of cigarettes	−0.140 (−0.319 to 0.039)	0.124	0.052 (−0.101 to 0.205)	0.507
Average waiting time of smoking first cigarette after waking up	0.014(−0.123 to 0.151)	0.839	0.181 (0.061 to 0.301) **	0.003
Harming themselves	0.056(−0.154 to 0.267)	0.601	0.159 (−0.015 to 0.333) ^†^	0.073
Harms on exposure to second-hand smoke	0.170(−0.035 to 0.375)	0.104	0.286 (0.119 to 0.453) ***	0.001
Adjusted R-square	0.055		0.123	
N	N = 390		N = 464	

Note: *** *p* ≤ 0.001, ** *p* ≤ 0.01, * *p* ≤ 0.05, and ^†^ *p* ≤ 0.1.

## Data Availability

The data presented in this study are available on request from the corresponding author.
